# Medical service utilization by children with physical or brain disabilities in South Korea

**DOI:** 10.1186/s12887-023-04309-2

**Published:** 2023-09-26

**Authors:** Sunyong Yoo, Ja Young Choi, Shin-seung Yang, Seong-Eun Koh, Myeong-Hyeon Jeong, Min-Keun Song

**Affiliations:** 1 Department of ICT Convergence System Engineering, Chonnam National University, Gwangju, Republic of Korea; 2 Department of Physical and Rehabilitation Medicine, Chungnam National University College of Medicine, Daegeon, Republic of Korea; 3 Department of Physical & Rehabilitation Medicine, Konkuk University School of Medicine, Konkuk University Medical Center, Seoul, Republic of Korea; 4 Department of Physical & Rehabilitation Medicine, Chonnam National University Medical School & Hospital, 160 baekseo-ro Dong-gu, Gwangju, 61479 Republic of Korea

**Keywords:** Medical service utilization, Children, Brain disability, Physical disability

## Abstract

**Background:**

Children with physical or brain disabilities experience several functional impairments and declining health complications that must be considered for adequate medical support. This study investigated the current medical service utilization of children expressing physical or brain disabilities in South Korea by analyzing medical visits, expenses, and comorbidities.

**Methods:**

We used a database linked to the National Rehabilitation Center of South Korea to extract information on medical services utilized by children with physical or brain disabilities, the number of children with a disability, medical visits for each child, medical expenses per visit, total medical treatment cost, copayments by age group, condition severity, and disability type.

**Results:**

Brain disorder comorbidities significantly differed between those with mild and severe disabilities. Visits per child, total medical treatment cost, and copayments were higher in children with severe physical disabilities; however, medical expenses per visit were lower than those with mild disabilities. These parameters were higher in children with severe brain disabilities than in mild cases. Total medical expenses incurred by newborns to three-year-old children with physical disorders were highest due to increased visits per child. However, medical expenses per visit were highest for children aged 13–18.

**Conclusion:**

Medical service utilization varied by age, condition severity, and disability type. Severe cases and older children with potentially fatal comorbidities required additional economic support. Therefore, a healthcare delivery system for children with disabilities should be established to set affordable medical costs and provide comprehensive medical services based on disability type and severity.

## Background

The World Health Organization (WHO) reported that over one billion people exhibit some form of disability, and approximately 15% of the world’s population experiences significant functional impairment [1]. Global disability prevalence is continuously rising due to population aging and increased chronic non-communicable disease incidence. The number of people with disabilities and comorbidities is also surging, intensifying the need for healthcare and rehabilitative services. Consequently, healthcare systems and families are increasingly financially burdened [2].

Stroke, cerebral palsy, traumatic brain injury, and other brain disabilities can paralyze the extremities, limiting daily life activities. Similarly, physical disabilities include amputation, joint disorders, and physical dysfunctions that impair daily life functions. These disabilities engender increased medical expenses and frequent medical services [3]; therefore, children with physical disabilities prompted by brain or physical disorders should be registered for customized medical services for better care. Factors that affect medical service use and expense for people with disabilities include demographics, such as age, gender, presence of a spouse, disability type, and severity; socioeconomic, including education and income level; health insurance; health status; and behaviors, such as the degree of necessary in daily life assistance. In particular, disability type, age, and behavior are closely linked to this medical service use and expenditure disparity [4].

The 2016 National Disability Survey announced that approximately 2,683,400 people with disabilities live in Korea [5]. According to the Korean Ministry of Health and Welfare, brain disabilities accounted for 14.0%, and physical disabilities for 5.1% of all children with disabilities in 2018. Furthermore, a 2014 survey by the Right to Health of Persons with Disabilities unit of the National Human Rights Commission of Korea revealed that many people with severe disabilities graded 1 to 3 did not attend regular medical check-ups due to imposed economic burden, doubts about medical treatments efficacy, poor hospital infrastructure, and a lack of convenient services. Notably, the primary obstacles encountered when seeking aid were inadequate medical personnel knowledge regarding disabilities, associated economic burden, poor facilities, insufficient rehabilitation centers and psychiatrists, information scarcity, and extensive wait times [6].

Rehabilitation for children with disabilities should focus on improving their development and achieving maximal functionality. In addition, rehabilitation should be fully accessible by integrating health systems at all levels and universal health coverage. WHO guidelines for health rehabilitation advocate for enhancing and expanding quality rehabilitation availability [7]. Of the 77 studies reviewed, one-third evaluated rehabilitative access barriers as secondary outcomes. Common barriers included logistic factors, such as distance and transportation costs; economic factors, including insurance, treatment, and service; inadequate knowledge; and attitudinal factors, such as service need and the lack of awareness. Moreover, many of these barriers are not restricted to people with disabilities. Specific disability-associated barriers included insufficiently trained healthcare providers, the lack of medical skills, communication barriers, and poor economic status [8].

Family income level impacts eligibility for national basic living security benefits that provide economic support to dependent family members. If a household is eligible for benefits and includes a family member with a disability, the government provides financial support and social welfare services; an allowance for a child with a disability is a disability-specific policy. Children younger than 18 with disabilities and who live at home receive economic support through a basic living subsidy [9]. However, existing policies are inadequate for those requiring varying medical and social support.

Furthermore, the existing medical system is currently inadequate [10]. Korean legislation on medical welfare promotes the rights of people with disabilities and encourages medical amenities, accessibility, awareness, and financial assistance improvements [6]. In addition, the current grading and registration system seeks to standardize medical and welfare services to those within the same disability grade [11]. Children with disabilities require different medical services relative to their developmental stage. For example, a previous study reported that children with disabilities had a higher prevalence of congenital complications, health declines, and a lower prevalence of infectious diseases than children without disabilities. Thus, these children require more medical services, increasing their hospitalization rates, comorbidities, and medical expenses [12]. Although epidemiological data on children with disabilities would illuminate how medical services should operate, such information is severely limited. Therefore, investigating their current situation is imperative for improving the prognosis of children with disabilities.

This study analyzed the medical visits, expenses, and comorbidities of children with physical or brain disabilities to investigate current medical service utilization in South Korea. This study establishes essential epidemiological information, such as the number of children with a disability, medical visits per child, medical expenses per visit, total medical treatment cost, and copayments. Additionally, medical service utilization relative to developmental stage and disability severity grade were analyzed.

## Methods

### Study area and duration

**Fig. 1:**
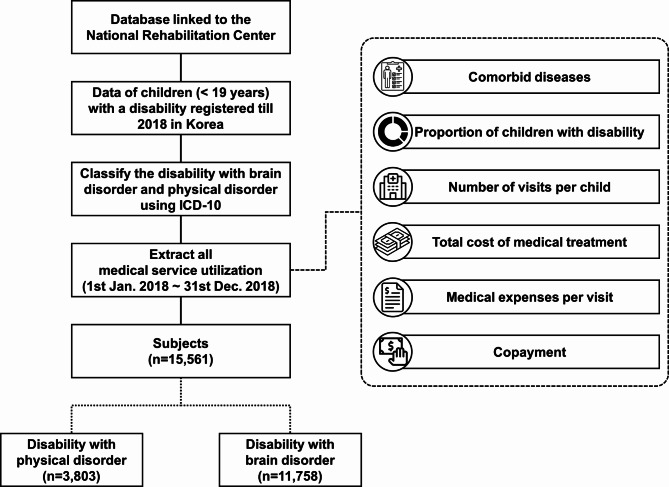
The flow chart illustrates the complete enumeration survey focused on children with brain and physical disorders

We reviewed the data of children with physical or brain disabilities aged 19 years or younger registered in the 2018 Korean Disability Registration and Grading System (Fig. 1); complete enumeration surveys for the subjects were included. All medical service utilization sources were extracted between January 1st and December 31st, 2018.

### Study design

Children with physical or brain disabilities incur more medical costs because of functional impairments and medical complications from their disorder than children with other diseases. Therefore, we evaluated the percentage of children with disabilities, medical treatments, visits per child, medical expenses per visit, overall treatment cost, and copayments.

### Disability definitions and severity

Medical service utilization quantifies or describes the services used to prevent or cure medical conditions. This study examined the medical services children received for their medical issues while living with a disability. We evaluated how many children had a disability, medical treatments, visits per child, medical expenses per visit, overall medical treatment cost, and copayments. Overall treatments for disease management quantified medical treatment occurrence and included medications, procedures, nursing, and rehabilitation. Medical visit prevalence denoted how many visits were made to clinics, and medical expenses per visit were calculated by dividing overall medical costs by the total visits. Overall medical treatment costs considered each medical expense incurred for treatment. Copayment was calculated using a predetermined rate at which patients pay for medical services upon treatment.

The Welfare of Persons with Disabilities Act delineates the clinical enrollment criteria for the Korean Disability Registration System [6], requiring that the Ministry of Health and Welfare Ordinance prescribes the disability degree. In addition, disabilities were justified in the Act on Welfare of Persons with Disabilities’ enforcement ruling [13].

Korea maintains a government-run grading and registration system for people with disabilities that focuses on physical and mental conditions. Disability severity is graded upon various criteria, allowing the government to incorporate people with disabilities into the welfare system. Korea recognizes 15 physical and mental disability types. Physical disabilities include brain, physical, renal, cardiac, respiratory, hepatic, or facial disorders; visual or hearing impairments; speech disabilities; intestinal or urinary fistula; and epilepsy. Intellectual disabilities such as autistic or psychological disorders are recognized as mental disabilities. This study focused on children with brain disorders categorized as disabilities from brain injury or disease, excluding those with mental or intellectual disabilities. In addition, we focused on children with physical disorders considered disabilities based on neuromuscular injury or disease.

Childhood diseases commonly diagnosed as brain disabilities are cerebral palsy, congenital hydrocephalus, brain anomalies, encephalopathy, meningoencephalitis, genetic disorders, multiple congenital anomalies, traumatic brain injuries, brain tumors, intracerebral hemorrhage, and other diseases related to brain disorders with functional impairments. These are classified as A83-87, B69, C70, C71, D32, D33, D42, D43, G00-08, G10, G11, G13, G35-37 G80-83, I60-69, P10, P52, P91, Q00-04, and R83 by the International Classification of Disease-10 (ICD-10) [14]. Alternatively, diseases commonly diagnosed as physical disabilities include congenital hypotonia, muscular dystrophy, spinal muscular atrophy, congenital myopathy, myelopathy, hereditary neuropathy, congenital arthropathy, osteochondral dysplasia, amputation, congenital agenesis of the limb, neuromuscular disease, brachial plexus injury, and other diseases related to neuromusculoskeletal disorder with functional impairments. These diseases are classified as C40, C41, C72, D16, D33.4, D36,1, G05, G06.1, G07, G08, G12, G13, G70-73, G83.4, G95, G98, G99, M00-99, Q05-07, Q65-79, and Q87 by the ICD-10 [14].

The Korean Disability Registration and Grading System judges the disability degree according to disability characteristics. For example, physical disability encompasses diseases in which bodily functions are permanently restricted due to various peripheral nervous and musculoskeletal system causations. Diagnosis is made when the disorder is alleviated after administering treatment specific to the condition. The standard period is six months after the disease or injury onset or more than six months of continuous treatment post-surgery. However, there are exceptions where disability fixation is apparent, such as amputation or spinal fixation. A physical examination, such as a manual muscle test, determines the overall dysfunction degree.

Brain disability is measured by the ability to walk and perform daily life activities while considering paralysis degree and extent, which is the main symptom. However, limb function deterioration due to spasticity, involuntary movements, balance disorders, and ataxia from brain lesions are also considered. Patients with cerebral palsy, stroke, brain damage, and other brain lesions are diagnosed with a disability after at least six months of continuous rehabilitation after onset. Children and adolescents can be diagnosed with a disability from age one or older, as determined by a doctor. Overall dysfunction degree is determined by physical examination, cognitive function evaluation, and a modified Barthel index; the Gross Motor Function Classification System, Gross Motor Function Measure, and Bayley’s Developmental Test are used for children aged 1–7 [13].

The 1998 Korean Disability Registration and Grading System classified disability into severity grades 1–6, based upon medical examination by disability type, to provide various welfare systems and support options. However, the disability grading system was abolished in July 2019 due to several emerging problems, such as the inability to provide adequate services tailored to individuals with disabilities. Disability severity grading now only operates within minimal divisions; grades 1–3 signify severe disabilities, whereas 4–6 indicate mild [15]. Therefore, this study classified subjects by disability severity: mild disability comprised those who could independently perform daily tasks, though they partly required personal assistance or assistive devices; severe disability included individuals highly dependent on personal assistance or assistive devices [11]. In Korea, the government provides different welfare services relative to disability severity.

### Sampling

This study achieved a complete enumeration survey for the subjects (disabilities = 15,561).

### Data collection and management

Data on medical service utilization by children with physical or brain disabilities were extracted from a database linked to the Health Care for the People with Disabilities (2019; National Rehabilitation Center), which included data from the National Health Insurance Service. Therefore, the data only entailed children’s medical services paid at medical clinics estimated by the National Health Insurance Service.

### Data analysis and interpretation

We statistically analyzed the proportion of children with a disability, visits per child, medical expenses per visit, overall medical treatment cost, and copayments by age group, severity, and disability type.

Differences among categorical variables, including severity and age, were tested using the chi-square test, and a *p*-value of less than 0.05 was considered statistically significant [16]. Next, the Jaccard index was calculated to analyze comorbidities between severe and mild physical or brain disabilities [17]. We statistically analyzed how much comorbidity overlap occurred in severe or mild diseases. The two terms’ co-occurrence was considered in the analysis; however, because the co-occurrence values do not consider individual term frequency, we used the Jaccard index as follows:

$$J\left(severe, mild\right)= \frac{\left|severe\cap mild\right|}{\left|severe\cup mild\right|}=\frac{{n}_{c}}{{n}_{o}}$$

We counted the co-occurring comorbidities (*n*_*c*_) in severe and mild diseases to calculate the Jaccard index. Then, the occurrence value (*n*_*o*_) was determined by the comorbidities in severe or mild diseases or both. For example, suppose 40 comorbidities occurred in severe cases, and 30 occurred in mild cases. If there were ten intersections between them with 50 aggregates, the Jaccard index is 10/50 or 0.2. The Jaccard index was calculated by dividing *n*_*c*_ by *n*_*o*_ for each disorder.

## Results

We analyzed the records of 3,803 children with physical disabilities and 11,758 with brain disabilities. First, we determined the disability severity and age groups among children with physical disabilities (Table 1). Children with a Grade 5 disability comprised the largest proportion (21.5%), followed by Grades 6 (19.5%) and 1 (17.7%). Among all children with physical disabilities, 55.2% exhibited a mild case. Those classified as Grade 1 incorporated the most male children (20.0%), whereas Grade 5 (25.1%) comprised the most females. The chi-square test confirmed a significant difference in disability severity between males and females (*p* < 0.05). Furthermore, the proportion of children with physical disabilities increased with age. Among children with physical disabilities, the largest age group was those aged 13 to 18 (65.1%). However, there were no significant age differences between males and females (*p* > 0.05). Next, we examined the severity and age groups among children with brain disabilities (Table 2). Children classified with a Grade 1 disability composed the largest proportion (57.9%), and among all children with brain disabilities, 79.9% were severe cases. The proportion of children with brain disabilities increased with age, and patients aged 13 to 18 were the most substantial (40.7%). There was a significant severity difference between males and females (*p* < 0.05), but none in age (*p* > 0.05).

**Table 1:** Disability severity and age groups among children with physical disabilities

			Total [n (%)]	Male [n (%)]	Female [n (%)]	
**Total**			3,803 (100.0)	2,273 (59.8)	1,530 (41.2)	
**Severity**	*Severe*	Grade 1	675 (17.7)	455 (20.0)	220 (14.4)	*Χ*^2^ = 36.839 *p*-value < 0.001
		Grade 2	372 (9.8)	240 (10.5)	132 (8.6)	
		Grade 3	657 (17.3)	397 (17.5)	260 (17.0)	
	*Mild*	Grade 4	540 (14.2)	322 (14.1)	219 (14.3)	
		Grade 5	820 (21.5)	436 (19.2)	384 (25.1)	
		Grade 6	741 (19.5)	424 (18.7)	317 (20.7)	
**Age (years)**	*Infant*	0–2	92 (2.4)	50 (2.2)	43 (2.8)	*Χ*^2^ = 7.191 *p*-value = 0.066
	*Toddler*	3–5	273 (7.2)	145 (6.4)	128 (8.3)	
	*Child*	6–12	961 (25.3)	585 (25.7)	376 (24.6)	
	*Adolescent*	13–18	2,478 (65.1)	1,494 (65.7)	984 (64.3)	

**Table 2:** Disability severity and age groups among children with brain disabilities

			Total [n (%)]	Male [n (%)]	Female [n (%)]	
**Total**			11,758 (100.0)	6,726 (57.2)	5,032 (42.2)	
**Severity**	*Severe*	Grade 1	6,811 (57.9)	3,880 (57.7)	2,932 (58.3)	*Χ*^2^ = 14.157 *p*-value = 0.028
		Grade 2	1,411 (12.0)	796 (11.8)	615 (12.2)	
		Grade 3	1,174 (10.0)	639 (9.5)	536 (10.6)	
	*Mild*	Grade 4	694 (5.9)	416 (6.2)	279 (5.5)	
		Grade 5	899 (7.6)	519 (7.7)	380 (7.6)	
		Grade 6	769 (6.5)	478 (7.1)	291 (5.8)	
**Age (years)**	*Infant*	0–2	893 (7.6)	521 (7.7)	372 (7.4)	*Χ*^2^ = 5.578 *p*-value = 0.134
	*Toddler*	3–5	2,226 (18.9)	1,226 (18.3)	1,000 (19.9)	
	*Child*	6–12	3,848 (32.7)	2,204 (32.8)	1,644 (32.7)	
	*Adolescent*	13–18	4,789 (40.7)	2,774 (41.2)	2,016 (40.1)	

Next, we assessed the medical service utilization of children with physical or brain disabilities (Table 3). Medical treatments, the number of children, total visits, total visits per child, and medical expenses per visit were recorded relative to disability severity. For both disability types, children with severe cases required more treatment and visits than those with mild disabilities, except medical expenses per visit in children with physical disabilities. Interestingly, among children with brain disabilities, those with a severe disability scored higher across all parameters than those with a mild disability, unlike our findings of those with physical disabilities.

**Table 3:** Medical service utilization by children with disability relative to condition severity

Type	Severity	Total medical treatments	Number of children	Total visits	Total visits per child	Medical expenses per visit ($)
**Physical disabilities**	*Severe*	80,298	1,800	92,877	51.598	77.06
	*Mild*	70,564	1,928	77,712	40.307	77.86
**Brain disabilities**	*Severe*	815,996	9,621	997,859	103.717	61.59
	*Mild*	129,985	2,056	140,409	68.292	35.74

# All costs were measured yearly

We also explored whether comorbidity rates differed between mild and severe disabilities by isolating the top 30%, resulting in more than 50 comorbidities, and comparing similarities using the Jaccard index. This analysis excluded comorbidities with too low a frequency since they acted as outliers or biases. We confirmed that the Jaccard index (*J*) of the comorbidities shared by those with mild or severe brain disabilities (*J*_brain_ = 0.063) was much lower than those of physical disabilities (*J*_physical_ = 0.137). These results indicate that the comorbidities of patients with brain disabilities significantly differ by severity, whereas comorbidities of patients with physical disabilities were relatively less different.

Next, we identified comorbidities in physical and brain disabilities that exhibited similar or large incidence rate differences between severe and mild cases (Table 4). Among comorbidities observed in children with physical disabilities, neuromuscular bladder dysfunction, tooth development or eruption disorders, vasomotor and allergic rhinitis, and dental caries exhibited significant differences in incidence rates. Alternatively, iron deficiency anemia, pain in the throat and chest, external ear disorders, lipoprotein metabolism and other lipidemias disorders, and heartburn incidence rates were similar. Among comorbidities observed in children with brain disabilities, epilepsy and recurrent seizures, pneumonia, convulsions, refraction and accommodation disorders, gastritis, and duodenitis demonstrated significantly different incidence rates. In contrast, superficial head injury, intracranial injury, other viral infections characterized by skin and mucous membrane lesions, cellulitis and acute lymphangitis, and other muscular disorders exhibited similar incidence rates.

**Table 4:** Comorbidities with similar or significant incidence rate differences between severe and mild cases of physical or brain disabilities

Type		Disease (ICD-10 code)	Number of children [n (%)]	
			Severe	Mild
**Physical disabilities**	*Different*	Pneumonia (J18)	222 (12.33)	129 (6.69)
		Neuromuscular bladder dysfunction (N31)	128 (7.11)	76 (3.94)
		Disorders of tooth development and eruption (K00)	238 (13.22)	345 (17.89)
		Vasomotor and allergic rhinitis (J30)	1194 (66.33)	1381 (71.63)
		Dental caries (K02)	290 (16.11)	458 (23.76)
	*Similar*	Iron deficiency anemia (D50)	51 (2.83)	51 (2.65)
		Pain in throat and chest (R07)	62 (3.44)	66 (3.42)
		Other external ear disorders (H61)	66 (3.67)	72 (3.73)
		Lipoprotein metabolism and other lipidemias disorders (E78)	62 (3.44)	63 (3.27)
		Heartburn (R12)	72 (4.00)	77 (3.99)
**Brain disabilities**	*Different*	Epilepsy and recurrent seizures (G40)	3,847 (39.99)	201 (9.78)
		Pneumonia (J18)	2137 (22.22)	232 (11.28)
		Convulsions (R56)	1487 (15.46)	137 (6.66)
		Refraction and accommodation disorders (H52)	1,657 (17.23)	602 (29.28)
		Gastritis and duodenitis (K29)	2,980 (30.98)	909 (44.21)
	*Similar*	Superficial head injury (S00)	287 (2.98)	61 (2.97)
		Intracranial injury (S06)	258 (2.68)	55 (2.68)
		Other viral infections characterized by skin and mucous membrane lesions (B08)	642 (6.67)	139 (6.76)
		Cellulitis and acute lymphangitis (L03)	520 (5.41)	113 (5.50)
		Other muscle disorders (M03)	327 (3.40)	72 (3.50)

Regarding medical service utilization, total visits per child with physical and brain disabilities decreased with age (Table 5). Younger children with disabilities require more medical services than older children due to frequent rehabilitative management. Medical expenses per visit also decreased with age, excluding ages 13 to 18 where costs rapidly increased due to the higher treatment costs for potentially fatal complications that arise as children with disabilities age.

**Table 5:** Medical service utilization by child age group with physical or brain disabilities

Type	Age (years)	Total medical treatments	Number of children	Total visits	Total visits per child	Medical expenses per visit ($)
**Physical disabilities**	Total	150,862	3,728	170,589	45.759	77.43
	*0–2*	2,421	24	2,947	122.792	84.16
	*3–5*	17,411	226	20,336	89.982	45.91
	*6–12*	49,606	932	52,739	56.587	34.25
	*13–18*	81,424	2,546	94,567	37.143	108.07
**Brain disabilities**	*Total*	835,832	9,986	995,416	99.681	58.16
	*0–2*	36,758	247	49,983	202.360	62.47
	*3–5*	259,666	1,945	331,284	170.326	58.34
	*6–12*	334,421	3,776	380,970	100.892	49.34
	*13–18*	204,987	4,018	233,179	58.034	71.40

# All costs were measured yearly

We also analyzed medical costs according to physical or brain disability severity (Table 6). The number of children, total treatment costs, total copayments, treatment costs per child, and copayments per child were recorded relative to disability severity. We found no differences in total treatment costs, total copayment, treatment costs per child, or copayments per child between severities in children with physical disabilities. However, in children with brain disabilities, the severe disability group exhibited higher total treatment costs, total copayments, treatment costs per child, and copayments per child than the mild disability group.

**Table 6:** Medical costs for caring for children with a disability relative to condition severity

Type	Severity	Number of children	Total medical treatment cost ($)	Total copayments ($)	Medical treatment costs per child ($)	Copayment per child ($)
**Physical disorders**	*Severe*	1,800	7,157,462.4	770,678.0	3,976.4	428.2
	*Mild*	1,928	6,050,837.9	767,989.6	3,138.4	398.3
**Brain disorders**	*Severe*	9,621	61,459,313.3	8,526,866.6	6,388.0	886.3
	*Mild*	2,056	5,017,996.0	1,102,057.3	2,440.7	536.0

# All costs were measured yearly

## Discussion

First, we analyzed the data of children with brain or physical disabilities through the Korean Disability Registration and Grading System, which defines disabilities quite differently than the Americans with Disabilities Act (ADA). ADA disabilities also include mental and physical medical conditions that can be easily determined as disabilities: amputation, attention deficit hyperactivity disorder, autism, bipolar disorder, blindness, cancer, cerebral palsy, deafness, diabetes, epilepsy, human immunodeficiency virus, intellectual disability (formerly termed mental retardation), major depressive disorder, mobility impairments (often requiring a wheelchair), multiple sclerosis, muscular dystrophy, obsessive-compulsive disorder (OCD), post-traumatic stress disorder (PTSD), and schizophrenia [18]. However, this means brain disability definitions differ between South Korea and the USA. Brain disability can appear as organic brain lesions, such as cerebral palsy, stroke, and traumatic brain injury, and can be combined with mental disabilities, such as intellectual disabilities and autism spectrum disorders. Therefore, we did not include mental disability in our assessment as this is a separate category of the Korean Disability Registration and Grading System.

The demographic data this study examined indicated a similar male-to-female ratio of children with a brain disability in South Korea. The male-to-female cerebral palsy ratio in the USA is approximately 1.5 to 1, indicating a notable difference between countries. We also determined that the total visits per child decreased with age in both physical and brain disabilities. Furthermore, medical expenses per visit decreased with age until reaching 13 to 18. Although hospital visits decreased in older children with disabilities, costs rapidly increased, likely due to the higher medical costs required to treat fatal comorbidities in aging children. Also, children with severe physical or brain disabilities required more treatments and visits than those with mild cases. However, only severe brain disabilities were associated with markedly higher total treatment costs, total copayments, costs per child, and copayments per child than the mild disability group. This finding may be because severe brain disabilities potentially require higher costs to treat more severe and fatal complications and comorbidities.

Brain disability comorbidities significantly differed by severity compared to physical disabilities, indicating a prominent and influential factor in medical service utilization differences between disability types. In particular, epilepsy, recurrent seizures, pneumonia, and convulsions frequently occur in severe brain disabilities, requiring increased medical use and expenses due to life-threatening complications and possible chronic diseases caused by brain lesions. Comorbidities in children with severe physical disabilities include pneumonia and neuromuscular bladder dysfunction, and treating these comorbidities raises medical costs.

Children in this population group had complex acute and chronic conditions; numerous and varied comorbidities, including cerebral palsy, congenital heart defects, and cancer; a broad range of mental health and psychosocial needs; major functional limitations; and a high mortality rate [19–22]. Additionally, the nervous system disorder class remained steady as children aged; these children had a chronic, cumulative profile due to healing difficulties and were predisposed to future complications in other systems [23]. A different comorbidity pattern existed in children with medical complexities and a high proportion of children in lower socioeconomic groups [24].

We also found that younger children with disabilities used medical services more than older children, but medical costs rapidly increased in older children with disabilities. The severe disability group was associated with higher total treatment costs, overall copayments, treatment costs per child, and copayments per child than the mild disability group. Medical expenses increased for older children with disabilities because more efforts and risks are apparent when treating potentially fatal comorbidities. In addition, medical costs may increase from secondary musculoskeletal problems, complications, or surgery during adolescence. However, the public medical insurance system in South Korea may have affected these results. Rehabilitation management was less costly for children below five than for older children, who required public medical insurance to support the increased medical costs. As evidenced in previous studies, caregivers may discontinue rehabilitative therapy as children with disabilities age because of the burden this places upon the family [3].

In a survey of families with children experiencing severe disabilities, Leonard et al. found that managing a spinal cord injury incurred the highest expenditure [25]. Using raw data from the 2006 National Living Condition Survey in Korea, additional costs for typical households with disabilities included ₩44,000 for people with mild and ₩185,000 for people with severe physical disabilities. Similarly, additional costs for severe brain disabilities were estimated at ₩152,000 [26]. Another Korean study further examined this data to determine how significantly additional costs aggravate poverty among households that included people with disability. After deducting the additional cost, poverty increased from 11.8 to 27.8%, and the additional cost of treating disabilities was confirmed as a prominent factor that aggravated poverty rates [27].

According to data on Korean children with disabilities gathered in 2011, the extra cost per household that included a child with a disability was approximately ₩455,000 per month. The extra expenditure in these households was primarily allocated to technical aids, education, and medical expenses. Independent variables, such as age, disability type, disability severity grade, and family income, also affected these additional costs. In particular, family income, disability grade, and the child’s age were the most significant [28]. However, data regarding these variables are severely lacking, and the information collected thus far varies substantially between physical or brain disabilities. Therefore, the data and conclusions from this study can support current data on the costs of caring for children with physical or brain disabilities.

Moreover, these findings are not isolated to South Korea. One study reported that depending on the disability severity, the United States of America exhibited a $77 to $80 income loss per month [29]. Another study indicated that seven working hours were lost per week [30]. Considering that the income lost for each day off work is substantial, this accumulates to an annual loss of $5,243 [31]. Many studies have explored age-per-cost profiles, but their results differ. Two studies published in the United Kingdom determined no significant differences in age-related expenditure or treatment frequency [32, 33]. However, another reported increased material costs for families with young children [34], while yet another indicated that parenting time was longer for toddlers than infants or preschoolers [35]. Thus, the burden can be significant for families caring for children with severe disabilities [36].

Disabilities that affect children and adolescents’ mental and physical health are correlated with many additional costs, especially for families with lower income [37]. According to a previous study on children with cerebral palsy, rehabilitation treatment rates sharply dropped after adulthood, attributed to the lack of rehabilitation hospitals [38]. Current rehabilitation treatment in adulthood targets those with severe disabilities, such as stroke and spinal cord injuries. Therefore, the system does not allow children with more mild disabilities to receive continuous health care when they are grown.

This study detailed and analyzed current medical service utilization for children with physical or brain disabilities in South Korea, evidencing the necessity for improved policies to support children with disabilities and their families. Notably, this study gathered data from the Korean Disability Registration and Grading System administrated by the Republic of Korea’s government and was the first to analyze medical service utilization by severity level in each disability. Some limitations included the focus on disability severity and age groups when evaluating how rehabilitation services should operate. In addition, various demographic factors such as family history, education level, and residential area characteristics were not sufficiently considered. Further studies should investigate the effects of comorbidities and combined disability in patients with physical or brain disabilities and consider outpatient and inpatient differences when evaluating medical services. With these improvements, our research can be a practical reference in establishing policies for providing medical services to children with physical or brain disabilities.

### Conclusion

Medical service utilization varies substantially by age, disability severity, and type. Children with severe disabilities and those that are older may potentially face fatal comorbidities and need more economic support. A healthcare delivery system for children with disabilities should be established to set affordable medical costs and provide comprehensive medical services based on disability frequency, type, and severity.

## Declarations

### Data and material availability

This study’s supporting data are available through the National Rehabilitation Center of the Ministry of Health and Welfare, Korea; however, restrictions apply. These data were used under license for the current study and are not publicly available. Data are available from the corresponding author (Min-Keun Song, drsongmk@jnu.ac.kr) upon reasonable request and with permission from the National Rehabilitation Center of the Ministry of Health and Welfare, Korea.
